# Tailored biosecurity training for veterinarians and farmers: bridging knowledge and practice gaps

**DOI:** 10.3389/fvets.2025.1643029

**Published:** 2025-10-27

**Authors:** Blerta Mehmedi, Jarkko Niemi, Claude Saegerman, Daniele De Meneghi, Anna Maria Iatrou, Ramazan Yildiz, Ilias Chantziaras, Alberto Allepuz, Ina Toppari, Georgios Batikas, Arvo Viltrop, Tarmo Niine

**Affiliations:** ^1^Department of Veterinary Medicine, Faculty of Agriculture and Veterinary, University of Prishtina, Prishtina, Kosova; ^2^Bioeconomy and Environment Unit, Natural Resources Institute Finland (Luke), Seinäjoki, Finland; ^3^Research Unit of Epidemiology and Risk Analysis Applied to Veterinary Sciences (UREAR-ULiège), Fundamental and Applied Research for Animal and Health (FARAH) Center, Faculty of Veterinary Medicine, University of Liège, Liège, Belgium; ^4^Department of Veterinary Sciences, University of Turin, Grugliasco-Turin, Italy; ^5^School of Agricultural Sciences, Department of Agriculture, University of Western Macedonia, Florina, Greece; ^6^Department of Internal Medicine, Faculty of Veterinary Medicine, Burdur Mehmet Akif Ersoy University, Burdur, Türkiye; ^7^Department of Internal Medicine, Reproduction and Population Medicine, Faculty of Veterinary Medicine, Ghent University, Merelbeke, Belgium; ^8^Department of Animal Health and Anatomy, Universitat Autònoma de Barcelona, Cerdanyola del Vallès, Spain; ^9^Animal Health ETT, Seinäjoki, Finland; ^10^Laboratory of Animal Production and Environmental Protection, School of Veterinary Medicine, Faculty of Health Sciences, Aristotle University of Thessaloniki, Thessaloniki, Greece; ^11^Institute of Veterinary Medicine and Animal Sciences, Estonian University of Life Sciences, Tartu, Estonia

**Keywords:** biosecurity training, veterinarians, farmers, knowledge gaps, practice gaps

## Abstract

Biosecurity is fundamental to animal health, public health, and the economic resilience of livestock systems; however, farm-level adoption remains uneven across regions. Knowledge gaps, language and financial constraints, and limited communication competence among veterinary advisers impede implementation, especially on small- and medium-scale farms. Behavior change-oriented interventions, such as Motivational Interviewing (which deploys multiple specific behavior change techniques as defined in BCTTv1), offer promise but are seldom embedded in veterinary curricula. This study proposes a concept and key elements for biosecurity training. It highlights a modular, evidence-based training framework developed under the COST Action CA20103 “BETTER” (2021–2025), aimed at improving biosecurity understanding and implementation by veterinarians and farmers. The initiative convened European experts to co-design a flexible curriculum that addresses both technical and behavioral challenges using participatory methods and interdisciplinary expertise. The resulting framework consists of five progressive modules: (1) Introduction, (2) Behavior Change and Communication, (3) Disease Transmission & Risk Assessment, (4) Emergency Response & Clinical Biosecurity, and (5) On-Farm Practices. These modules are designed to be combined in a “pick-and-choose” format to match local needs, target audiences and resources. Delivery blends online micro-lessons, participatory workshops, peer networks, and low-cost on-farm demonstrations, while materials are culturally and linguistically adapted and framed in terms of clear economic benefits. Continuous feedback loops encourage iterative refinement and habit formation during the learning process. The proposed training framework seeks to transform biosecurity from a prescriptive doctrine into a farmer-owned daily routine by integrating technical content with behavioral science and context-specific delivery.

## Introduction

Effective biosecurity measures are essential for ensuring animal health, food security, public health, financial sustainability of farming operations, and global trade (1). Despite their importance, these practices are often under-implemented or inconsistently applied at the farm level (2). Although theoretical knowledge has improved, a substantial gap remains in the farm-level implementation of these measures (3, 4).

Although veterinarians are considered key actors in educating farmers on biosecurity, their efforts are hampered by limited insight into the existing knowledge of both farmers and fellow practitioners (5, 6). This reflects a broader limitation of the traditional knowledge transfer approach, which often assumes a unidirectional flow of information from expert to farmer. However, this deficit model overlooks the fact that farmers are not passive recipients of advice but active actors with rich experiential knowledge influenced by their experience, local context, networks, and values. This new information must be integrated into their existing beliefs, attitudes, and situational constraints. Additionally, time constraints and differing priorities further complicate their ability to support biosecurity implementation (7). This highlights the need for improved engagement strategies to encourage compliance among farmers (8, 9).

Furthermore, many farmers, particularly those on medium-sized farms and smallholders, face financial constraints that limit their ability to implement these measures on their farms (8). Language represents a training barrier affecting farmers’ access to biosecurity information, making it especially difficult to acquire crucial knowledge in their native language. This challenge is particularly relevant for farmers who may not be proficient in the dominant language used in training materials or in communication with veterinarians (10). Language issues concern, on one hand, training when the target groups speak different languages, which can also be the case in multicultural workplaces, and on the other hand, the level of “technicality” and understandability of training materials. To improve farmers’ understanding and adoption of biosecurity measures, addressing these language barriers through tailored communication strategies and multilingual training resources is essential. This will ultimately contribute to improved animal health and disease prevention (35). Another important obstacle is the economic barriers to biosecurity. These barriers include structural financial limitations (e.g., low or unpredictable farm income) and behavioral factors such as present bias (the tendency to prioritize short-term cost savings over long-term benefits) (8). This is especially problematic for medium- and small-scale farmers, who have limited financial resources and require cost-effective strategies to ensure sustainability and compliance (11, 12). Financial constraints hinder their ability to adopt necessary biosecurity measures, as many small-scale farms struggle to allocate the funds required for these practices (13, 14). In contrast, farmers with larger herds or privately owned land tend to adopt these practices (13, 15). This indicates that medium- and small-scale farmers need adapted biosecurity measures and financial support to effectively implement these measures, thereby ensuring their sustainability and compliance (15).

Despite being aware of best practices, many farmers misunderstand biosecurity measures, leading to inconsistent implementation as research shows (3, 16). These challenges must be proactively addressed to ensure the long-term effectiveness and on-farm adoption of biosecurity programs. Small-scale farming operations often lack access to comprehensive information on animal health, resulting in suboptimal biosecurity practices (36). Additionally, there is a gap between farmers’ perceptions of biosecurity and their actual practices, highlighting that positive attitudes do not always translate into effective implementation (13). Buckel et al. (8) similarly observed that awareness alone may not translate to consistent on-farm practices. This phenomenon, commonly referred to as the attitude-behavior gap, underscores the importance of moving beyond technical knowledge to address motivation, habits, and social influences. This attitude-behavior gap poses risks to livestock and human health. Moreover, the effectiveness of biosecurity enforcement may be hindered by farmers who do not fully trust the advice of their veterinarians (1). Traditional training programs have been criticized for their limited and short-term efficacy. Although they may initially ensure compliance, their effectiveness tends to diminish over time, without ongoing support. In contrast, customized coaching methods have the potential to enhance farmers’ long-term compliance with biosecurity measures (9). Such methods, especially those grounded in behavioral science, seek to build intrinsic motivation and shared decision-making rather than simply delivering information. One such approach is Motivational Interviewing (MI), which uses open-ended dialogs, active listening, and goal setting to explore a farmer’s reasons for change.

To address these challenges, the COST Action CA20103 BETTER (Biosecurity Enhanced Through Training Evaluation and Raising Awareness) was launched to evaluate current biosecurity practices and identify key motivators and barriers to implementation using a participatory approach. The initiative aims to expand biosecurity training among veterinarians and farmers and to develop evidence-based communication strategies tailored to the diverse needs of stakeholders across Europe and beyond.

To enhance the practical application and contextual relevance of the biosecurity training curriculum, its development followed a structured participatory process involving experts from various disciplines and regions. The expert team comprised professionals from the fields of veterinary epidemiology, public health, behavioral science, veterinary education, and animal health economics. This interdisciplinary foundation ensured the integration of technical knowledge, pedagogical strategies, and behavior change expertise throughout the training framework. Although the geographical scope was primarily pan-European, the modules were designed for scalability and contextual adaptation in LMICs, where institutional support, digital infrastructure, and language access may be limited. The modular structure allows localization based on resource constraints, cultural expectations, and learner profiles. At this stage, the modules have not been formally validated or pilot tested. Instead, the BETTER initiative concentrated on co-developing a ready-to-implement framework that will serve as a foundation for future testing and refinement. Planned future steps include evaluation, field piloting, and feedback collection from key stakeholders to assess usability, contextual appropriateness, and training effectiveness. The final training package reflected a synthesis of scientific evidence, pedagogical principles, and practical field experience. It places significant emphasis on behavior change strategies, multilingual accessibility, and flexible delivery formats (including digital and non-digital formats). These features were intentionally designed to support future adaptation and rigorous evaluation, with the ultimate goal of promoting long-term improvements in biosecurity-related behaviors among farmers and veterinary professionals. This study addresses a critical gap in formal biosecurity education, which has long hindered the adoption of sustainable practices. The urgent need for tailored, modular biosecurity training programs has been increasingly recognized (17). This recognition emerged from a series of face-to-face expert meetings held between 2021 and 2025 as part of the BETTER project, where participants combined insights from technical risk assessment and behavior change science to co-design the framework. Rather than applying a formal curriculum-design framework, the working group employed a participatory co-design approach, convening a two-day in-person workshop to brainstorm and agree on the curriculum outline. The resulting training programs prioritize practical applicability, accessible communication, and sustainability. They are designed to support local trainers, veterinarians, and extension workers in implementing evidence-based and flexible approaches tailored to the biosecurity needs of diverse farming systems.

This study proposes a concept and key elements for biosecurity training. It highlights a modular, evidence-based training framework developed under the BETTER initiative, aimed at improving biosecurity understanding and implementation among veterinarians and farmers. The outcome of this perspective is a flexible training outline that can be adapted based on local needs, target audiences, and available resources, thereby contributing to more resilient and sustainable livestock farming practices.

## Strengthening competence/communication skills in animal biosecurity among veterinary practitioners

Veterinarians are competent professionals who promote animal health and welfare, prevent disease, ensure food safety, and protect human health within the One Health framework. Their responsibilities extend beyond their professional expertise. They must possess interpersonal, advisory, and communication skills to translate their biosecurity knowledge into effective farm practices. To fulfill these roles effectively, veterinarians must continually develop their technical and communication skills, as well as behavior change methodologies tailored to diverse farm contexts (Table 1).

**Table 1:** Proposed biosecurity training framework as models for veterinary professionals and paraprofessionals (VPP) and farm personnel (FP).

Category	For veterinary professionals & paraprofessionals (VPP)	For farm personnel (FP)
Module 1 — Introduction		
Training Objectives	· Understand the definition, scope, and public health/One Health link of biosecurity. · Understand basic biosecurity principles and their importance. · Identify common biosecurity risks and transmission pathways in animal health. · Recognize the added value of biosecurity.	· Understand the “What & Why” of Biosecurity (“*Biowhat*?”) in simple terms. · Grasp core biosecurity principles. · Understand why biosecurity matters in a farm setting.
Training Approach	· Online courses, video lectures, in-person lectures. · Online modules & webinars. · Multiple-choice question tests.	· Online courses, video lectures, in-person lectures. · Online modules & webinars. · WhatsApp/SMS micro-lessons, video tutorials, mobile apps.
Key Considerations and Other Aspects	· No prior knowledge about biosecurity required. · Flexible timing (on-demand).	· Simple, clear language—avoid jargon. · No prior knowledge about biosecurity required. · Flexible timing (on-demand).
Module 2 — Communication		
Training Objectives	· Develop strategies for engaging farmers on biosecurity. Demonstrate empathy and professional humility toward farmers’ lived realities; practice shared decision-making. · Understand regulatory/legal aspects and behavior-change strategies. · Improve biosecurity compliance by applying the Capability-Opportunity-Motivation Behavioral (COM-B) model and Behavior Change Techniques. Develop skills in MI and other evidence-based communication techniques.	· Learn peer-sharing techniques. · Share biosecurity practices effectively within the farm team and community to bring about change. · Understand the economic and behavioral incentives that shape decisions around the biosecurity compliance.
Training Approach	· Interactive discussions (e.g., World Café, focus group). · Training in behavior change framework, including: COM-B, Behavior Change Wheel. · Communication skill-building through: motivational interviewing (OARS - Open questions, Affirmations, Reflective listening, and Summary reflections) and other health communication techniques. Map barriers and select intervention functions using COM-B/BCW (capability (C), opportunity (O), and motivation (M)/Behavior Change Wheel (BCW) and specify techniques using the BCTTv1 taxonomy (e.g., action planning, prompts/cues, habit formation, social support).	· Mentorship programs with experienced peers. · Peer-exchange visits. · Farmer storytelling (loss & recovery narratives). · Group discussions (“Farmers’ clubs”). · Role-play for difficult conversations and trust-building
Key Considerations and Other Aspects	· Focus on transforming from advisor to behavioral coach, grounded in autonomy support and mutual respect. · Networking for knowledge sharing.	· Networking for knowledge sharing. · Considerations for adaptation in multicultural, multilingual, and LMIC (Low- and Middle-income Countries) settings, with inclusive facilitation techniques.
Module 3 — Risk assessment		
Training Objectives	· Differentiate between zoonotic and non-zoonotic disease transmission pathways. · Analyze high-risk scenarios for disease introduction and spread, considering economic impacts. · Develop a foundational biosecurity risk assessment for a specific farm setting.	· Understand how pathogens spread: when considering new animals, quarantine, people, equipment, environment, wildlife and pests. · Recognize farm-specific disease risks and common transmission routes. · Understand how biosecurity reduces disease losses and increases productivity.
Training Approach	· Analysis of case studies of major outbreaks (e.g., African Swine Fever, foot-and-mouth disease, Avian Influenza). · In-depth risk assessment exercises and economic impact modeling. · Identifying common biosecurity risks and transmission pathways relevant to animal health scenarios.	· Quick case studies (e.g., impact of visitor limits, effectiveness of footbaths). · Practical exercises to identify farm-specific risks. · Discussions on applying simple, cost-effective biosecurity measures in daily workflow.
Key Considerations and Other Aspects	· Survey associations for priority topics for advanced content. · Pre-course baseline assessment (by using, e.g., Mentimeter, photo/video) to tailor content. · Post-course feedback for continuous adjustment.	· Tailor content and methods based on farm type and local context. · Emphasize simple, actionable steps. · Post-course feedback for continuous adjustment.
Module 4 — Emergency response		
Training Objectives	· Master outbreak investigation, reporting, containment, and depopulation protocols. · Understand interagency collaboration during emergencies. · Conduct biosecurity audits and surveillance. · Implement clinical biosecurity: infection control (PPE (personal protective equipment), hand hygiene), isolation of contagious animals, proper biological waste handling/disposal.	· Implement key daily biosecurity habits. · Understand the value of prevention vs. treatment (success stories). · Recognize early warning signs of disease (early-signs checklist). · Know who to contact in case of a suspected outbreak (reporting protocols). · Understand basic isolation success strategies.
Training Approach	· Online modules & webinars for theoretical aspects. · Hands-on workshops & simulations for outbreak scenarios and PPE use. · Case-based problem-solving for complex situations. · Develop, implement, and audit biosecurity protocols in diverse settings.	· Develop practical tools: “5-Minute Biosecurity Check,” “3-rule farm plan,” “Minimal viable biosecurity plan.” · Case studies illustrating successful isolation or prevention. · Interactive sessions on spotting early signs and reporting procedures.
Key Considerations and Other Aspects	· Quizzes & certification exams to validate competency. · Focus on regulatory compliance and official procedures. · Post-course feedback for continuous adjustment.	· Content directly relevant to daily farm operations. · Quizzes or practical demonstrations of understanding. · Post-course feedback for continuous adjustment.
Module 5 — On-farm practices		
Training Objectives	· Design and advise on comprehensive on-farm biosecurity plans (entry controls, hygiene, visitor management, animal movement, quarantine, disinfection). · How to train farm personnel on implementing specific on-farm biosecurity measures. · Audit and evaluate the effectiveness of on-farm biosecurity practices.	· Implement effective entry controls, hygiene protocols, and visitor management. · Manage animal movement, quarantine, and disinfection procedures correctly. · Implement “Easy Measures”: vaccinations, clean water and feeding, footbaths, logbooks, vehicle and equipment washing, manure and waste disposal, rodent control. · Contribute to and follow a specific biosecurity plan for their farm.
Training Approach	· Develop template biosecurity plans for various farm types. · Lead practical exercises and demonstrations in field conditions. · Facilitate case-based problem-solving for specific on-farm challenges. · Conduct hands-on workshops & simulations for implementing and auditing practices.	· Hands-on farm demonstrations of all key practices. · Practical exercises in real or simulated farm conditions. · Use of real-world case studies and practical examples. · Development of farm-specific checklists and protocols.
Key Considerations and Other Aspects	· Requires an in-person, hands-on approach for effective training and auditing. · Focus on tailoring advice to specific farm contexts and resources. · Least flexible module due to its practical nature.	· Requires an in-person, hands-on learning approach. · Emphasize practicality and integration into daily routines. · Least flexible module; strong emphasis on practical application on the farm.

Module 1: Introduction to Biosecurity: Overall Goal and Ranking: Fundamental information; Easiest and least resource-intensive; Passive learning options. Primary Target Groups: VPP, FP. Module 2: Behavior Change and Communication: Overall Goal and Ranking: Skill-building; Focus on behavior change, communication, and stakeholder engagement. Primary Target Groups: VPP (specifically VP (veterinary practitioner), AHP (Animal Health Partners), GV (Government Veterinary Services), FP (farm personnel)), (specifically Farmers, Managers). Module 3: Disease Transmission & Risk Assessment: Overall Goal and Ranking: Intermediate; Builds on Module 1; Focus on understanding disease spread and practical risk evaluation. Primary Target Groups: VPP (specifically VP, GV), FP (Farmers, Managers, Association Members). Prerequisite: Module 1 (Introduction to Biosecurity) knowledge and understanding. Module 4: Emergency Response and Clinical Biosecurity: Overall Goal and Ranking: Advanced; Focus on preparedness, outbreak management (VPP), and daily vigilance (FP). Primary Target Groups: VPP (specifically VP, GV), FP (All farm personnel). Prerequisite: Recommended: Modules 1 & 3. Module 5: On-Farm Practices: Overall Goal and Ranking: Practical Application; Most resource-intensive (time, hands-on work); Focus on implementing tangible biosecurity measures. Primary Target Groups: FP (All farm personnel – primary implementers), VPP (Advisory, training, auditing role). Prerequisite: Recommended: Modules 1 & 3. Module 4 for those involved in emergency plan components.

An important prerequisite for behavior change is the establishment of empathy, trust, and shared decision-making between the practitioner and the client (18). This can be achieved through approaches such as MI, which is defined as an intervention to promote “change talk” among farmers’ expressions, willingness, and motivation to change, which correlates with a stronger commitment to biosecurity measures (19–23). The benefits of MI are reinforced by qualitative assessments that advocate the integration of evidence-based communication strategies into veterinary education (19). Notably, substantial improvements in communication competence have been observed among cattle veterinarians who have received MI training, facilitating more effective herd management (23). Even brief MI training sessions have shown positive effects, with increased client participation in herd health discussions (19, 20, 22). Despite these advantages, there is a need for more client-centered approaches to meaningfully influence farm decision-making.

In addition, veterinary education must adopt interdisciplinary strategies that combine behavioral science, epidemiology, and economics to equip veterinarians with the tools to provide contextually and credible sound advice. Biosecurity communication should align with farmers’ realities and perceptions to promote sustainable changes. In addition to communication, technical biosecurity competence is essential. Veterinarians must be proficient in farm-specific risk assessment, emergency response, hygiene protocols, and biosecurity audits. Veterinarians trained in structured risk evaluation and outbreak simulations can improve biosecurity compliance among farmers, particularly in the case of notifiable diseases (12, 15), emphasizing the role of harmonized assessment tools in ensuring a consistent biosecurity evaluation and guidance (37).

Despite the importance of communication skills, gaps persist in veterinary education, including limited dedicated coursework on communication theory, minimal use of experiential learning methods, and a lack of structured assessment and feedback on communication competencies (38). Many practitioners have reported feeling unprepared to work in this field of expertise (38). To respond effectively to evolving biosecurity threats and standards, veterinary training must evolve to emphasize adaptive communication and technical proficiency. Programs that incorporate behavioral science and communication theory equip veterinarians to deliver contextually relevant and evidence-based recommendations to increase the personal motivation of farmers to commit to behavior change by inspiring them to adopt (24, 25).

## Empowering farmers through accessible biosecurity education

Farmers often lack an enabling environment and the necessary incentives for the sustained adoption of biosecurity practices. Traditional passive methods, such as lectures, printed materials, and one-time seminars, are inadequate for generating long-term behavioral changes (1). In contrast, participatory and applied learning models, such as on-farm workshops, peer-to-peer exercises, and success stories, provide real-time feedback and hands-on demonstrations, making biosecurity concepts more memorable and practical (4, 9, 26). However, the effective implementation of these approaches requires time, commitment, and resources. Simultaneously, digital and visual tools are transforming biosecurity training, particularly for dispersed or low-literacy farming communities in remote areas. Mobile apps, WhatsApp micro-lessons, and video tutorials can deliver bite-sized content and allow performance benchmarking using risk-mapping dashboards (10, 27, 28, 36). To maximize their spread and understanding, materials must be culturally and linguistically adapted using native languages, simplified terminology, and locally relevant narratives (4, 29–31). Economic framing can enhance farmers’ engagement in biosecurity, particularly when training quantifies the benefits (reduced losses, treatment savings, and productivity gains) relative to costs (12, 15, 32). Additionally, collective learning environments fostered through networks and cooperatives enhance information exchange and commitment to biosecurity goals (7, 39). Finally, fostering habit formation through behavior change interventions, such as “5-min biosecurity audits” or farm-specific checklists, ensures that biosecurity becomes an integral part of daily operations (33).

**Figure 1:**
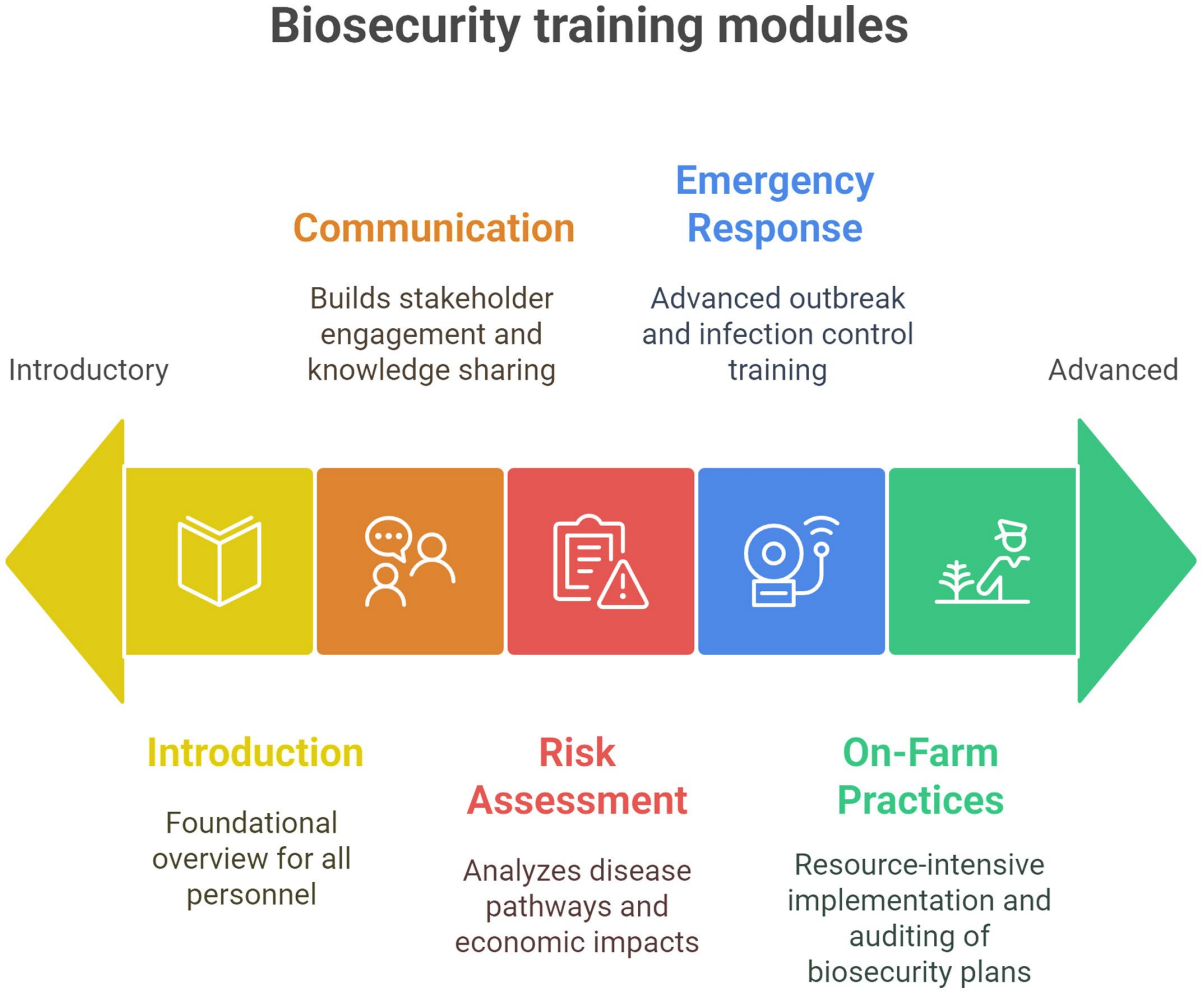
Biosecurity training modules ordered by implementation difficulty (left: easier → right: harder).

In line with the previous sections, we propose a biosecurity framework for veterinary practitioners and farmers (Table 1; Figure 1).

General remarks:

- This framework offers modules in a “pick-and-choose” style.
- Modules are generally presented starting from the easiest and least resource-intensive (Module 1) to the most complex and resource-intensive (Module 5).
- Course designers should select and adapt modules based on the specific target audience, the learning objectives, and the available resources.
- VPP: Veterinary Professionals (Veterinary Practitioners, Regulatory and Government Veterinarians) and Paraprofessionals (Veterinary Students and other Animal Health Professionals, such as technicians).
- FP: Farm Personnel (smallholder and commercial livestock farmers, farm managers and supervisors, farm workers and caretakers, and members of farmers’ and producers’ associations).
- PPE: Personal Protective Equipment.

## Discussion

The training framework outlined in this study emphasizes the need for biosecurity education that is both targeted and adaptable. This approach involves diversifying content(s) for veterinarians and farmers while utilizing flexible delivery methods, such as messaging platforms, mini-surveys, in-person demonstrations, and practical exercises in field conditions. Importantly, training should be cyclical (iterative process) and critical, incorporating ongoing mentorship, feedback, and refinement of strategies to ensure responsiveness to evolving risks and the needs of farmers. The persistent gap between biosecurity knowledge and its practical application on farms highlights the urgent need to change the manner in which biosecurity training is delivered to veterinarians and farmers. This perspective underscores the importance of tailored, context-specific education that addresses the real-world challenges and barriers faced by the stakeholders. As veterinary professionals and farmers work within a shared ecosystem of disease prevention, it is vital to enhance their respective competencies and communication channels to support sustainable and effective biosecurity. This is one of the reasons why we presented the data in Table 1 with veterinary practitioners and farmers in parallel.

Traditional biosecurity training programs typically adopt a top-down approach, focusing on information dissemination rather than participatory engagement. Although such an approach may establish baseline awareness, it often fails to promote behavioral change and long-term compliance (1). Increasing evidence supports the effectiveness of interactive and context-driven methods, such as on-farm workshops, visual aids, motivational interviewing, and peer learning networks, in enhancing the retention and practical application of biosecurity principles (9, 19). This aligns with practical learning models and reflects a growing consensus within veterinary pedagogy that highlights learning by doing, especially in areas where theoretical training does not readily translate into behavioral change. Tailored communication and training strategies are essential for addressing the diverse needs of farmers, who may encounter barriers such as limited literacy, economic constraints or language difficulties (4, 29). However, it is important to avoid generalizations that assume that all farmers require only simplified information. In reality, the farming community is highly heterogeneous and comprises individuals with varying levels of expertise, education, and interests. While some farmers may benefit from basic and accessible guidance, others possess advanced knowledge and seek in-depth, evidence-based content that allows them to engage with and implement complex biosecurity measures. This variation underscores the need for differentiated training approaches that respect and reflect the diversity of the farming population. Moreover, a significant challenge lies in reaching farmers who are most in need of biosecurity education but are often the least likely to access conventional training. Addressing this gap is critical to ensuring the widespread and effective adoption of biosecurity measures in agricultural systems worldwide. An important barrier to effective implementation is the cost and perceived burden of biosecurity interventions, especially for small- and medium-scale farmers. Unlike large-scale operations, which often have better access to veterinary services and infrastructure, resource-constrained farmers require low-cost and scalable solutions that fit their daily routines. Therefore, a “minimal-viable-biosecurity” model focusing on essential practices such as controlled animal movement and purchase, safe entry, vaccination, clean water supply, and food waste and by-product management may serve as a pragmatic entry point for adoption by the industry. Simple tools, such as the “5-Minute Biosecurity Check” or stepwise plans, can help foster habit formation, which is crucial for sustaining long-term changes. These tools must be designed for easy implementation, allowing users to repeat specific behaviors until they become automatic (33). By simplifying the process of adopting biosecurity measures, these tools can help overcome barriers, such as time constraints and complexity, which often hinder compliance (34).

Ultimately, the future of animal health management depends on integrated, human-centered, and biosecurity training model development. By combining technical knowledge with behavioral insights, economic realities, and local languages, we can promote a more inclusive and effective approach to disease prevention in the future. This requires both improved content delivery and strategic policy support to embed biosecurity training into broader animal health and educational frameworks. Moving forward, enhanced collaboration among veterinary educators, policymakers, farmer organizations, and animal health professionals is required to translate knowledge into sustainable actions to improve farm resilience. Tailoring the biosecurity training according to the local context, available resources, and farmer’s needs is a necessary investment, and the current perspective aims to provide a backbone of modules where stakeholders can pick and choose parts of the training to include into the training with the principle of “fit-to-purpose.” This modular structure not only supports adaptability across different contexts but also offers a path toward harmonized, competency-based approaches to biosecurity training that can be recognized across EU and non-EU education systems.

## Data Availability

The original contributions presented in the study are included in the article/supplementary material, further inquiries can be directed to the corresponding author.
